# Beak fracture associated with leiomyosarcoma in a budgerigar (*Melopsittacus undulatus*): a case report and literature review

**DOI:** 10.3389/fvets.2023.1309185

**Published:** 2023-12-08

**Authors:** Brittany L. Rasche, Daniel Felipe Barrantes Murillo, Tatiane Terumi Negrão Watanabe

**Affiliations:** ^1^Department of Population Health and Pathobiology, College of Veterinary Medicine, North Carolina State University, Raleigh, NC, United States; ^2^Department of Diagnostic Medicine/Pathobiology, College of Veterinary Medicine, Kansas State University, Manhattan, KS, United States; ^3^Department of Pathobiology, College of Veterinary Medicine, Auburn University, Auburn, AL, United States; ^4^Antech Diagnostics, Los Angeles, CA, United States

**Keywords:** budgerigar, *Melopsittacus undulatus*, leiomyosarcoma, beak, neoplasia

## Abstract

A 2-year-old male budgerigar (*Melopsittacus undulatus*) died after a 1-day history of fracture of the rostral rhinotheca with pale mucous membranes, dyspnea, dull mentation, and ataxia. Histopathology revealed an infiltrative neoplasm composed of interweaving streams of spindle cells effacing the dermis and bone of the rostral upper beak as well as a ganglion and two cranial nerves. No visceral metastasis was observed. Neoplastic cells exhibited strong cytoplasmic immunolabeling for alpha-smooth muscle actin (α-SMA) and lacked immunolabeling for S100, Melan-A, PNL2, and cytokeratin AE1/AE3. These findings were consistent with a locally invasive leiomyosarcoma Leiomyosarcomas arise from the smooth muscle and are locally invasive with rare metastases. In birds, leiomyosarcomas are mostly reported to arise from the spleen, gastrointestinal, and reproductive tracts. In the case report herein, we describe the histological and immunohistochemical features of a primary beak leiomyosarcoma in a budgerigar associated with a fracture located at the rostral rhinotheca. Leiomyosarcoma arising from the beak has not been described in the literature.

## 1 Introduction

In birds, fractures of the beak are most related to trauma. However, underlying diseases, such as local infection, malnutrition, metabolic disorders, chronic hepatic disease, or neoplasia, can weaken the beak, making it more susceptible to fracture with minor trauma (pathologic fracture) (1).

Neoplasms of the beak serve as one possible cause of pathologic beak fracture and can also predispose to secondary bacterial and fungal infections, which further contribute to weakening of the beak. In birds, especially budgerigars and cockatiels, fibrosarcoma is considered the most common primary beak tumor (1). Fibrosarcomas may cause deformation of the beak by invasion of the nares, nasal turbinates, and associated bones (1). Other common primary beak tumors include squamous cell carcinoma and malignant melanoma (1). Metastatic tumors also have the potential to invade and efface the beak but are considered less common. A summary of clinical findings, tumor location, and immunohistochemistry profile (when performed) for several selected case reports of primary beak neoplasms in a variety of bird species are presented in Table 1 (2–14). Additionally, neuroma formation following partial beak amputation has been reported in chickens (15). In the case report herein, we describe the histological and immunohistochemical features of a primary beak leiomyosarcoma causing a rostral rhinothecal fracture in a budgerigar. Leiomyosarcoma arising from the beak of birds has not been previously described in the literature.

**Table 1:** Selected cases of neoplasia arising from the beak anatomical locations.

Species	Age	Sex	Tumor	Location	IHC	Cause of death	References
*Psittacis erithacus*	12 y	NS	Widespread sarcoma (primary site undetermined)	Proximal left tarsometatarsus Upper beak	NA	Pathological fractures at the upper beak and left tarsometatarsus below the articular cartilage	(2)
*Eudyptes chrysolophus*	NS	f	Malignant melanoma	Necrotic and caseous mass effacing the cere, rostral sinuses, face, eyes, and upper beak	NA	Euthanasia, severe progressive weight loss, and metastases to the underlying skeletal muscle and adrenal glands	(3)
*Psittacus erithacus erithacus*	8 y	NS	Malignant melanoma	Upper beak deformed and protruded, black mass effacing nasal cavity, pancreas, ovary	NA	Euthanasia, metastases to the carotid artery, intestine ovary, and pancreas, fungal culture sinuses *Candida krusei*	(4)
*Ara ambigua*	28.5 y	f	Squamous cell carcinoma	Rostral left mandibular beak	NA	Died after 30 weeks of treatment, anorexic without evidence of metastases	(5)
*Answer anser*	18 mo	m	Xanthomatous cutaneous neoplasia	Submandibular mass, feet and periocular skin	Vimentin (−) Lysozyme (−) S-100 (−)	NS lost follow-up	(6)
*Melopsittacus undulatus*	6 y	m	Kerathoacanthoma	Maxillary beak	NA	Euthanasia	(7)
*Columba livia*	12 y	m	Malignant melanoma	Lower beak	NA	Stopped eating and died 7 months after the initial presentation	(8)
*Rynchopsitta pachyrhyncha*	NS	m	Malignant melanoma	Left mandibular beak	NA	Died after 11.5 weeks of radiation therapy and lung metastases confirmed during necropsy	(9)
*Eudyptes chrysolopus*	NS	NS	Malignant melanoma	Below skin right cere	S-100 (−) Melan-A (−) PNL-2 (+) HMB-45 (−)	Died after 39 months post-diagnosis, with no evidence of metastases at biopsy time	(10)
*Psittacus timneh*	34 y	m	Squamous cell carcinoma and penicillin-resistant *Staphylococcus pseudointermedius*	The left side of the rhinotheca	NA	Died 7 months after initial diagnoses and 4 months after radiation therapy, with no visible tumor regrowth	(11)
*Cacatua alba*	10 y	f	Infraorbital keratin cyst	The right commissure of the beak	NA	No recurrence of the mass 1 year after surgical excision	(12)
*Pionites melanocephalus*	8 y	m	Squamous cell carcinoma	Right gnathotheca	NA	9 months after the initial evaluation the bird was in a good quality of life	(13)
*Ara chloropterus*	22.5 y	f	Squamous cell carcinoma	Right gnathotheca	CK AE1/AE3 (+)	Euthanasia due to extension of the lesion after 16 months	(13)
*Melopsittacus undulatus*	10 y	m	Kerathoacanthoma and *Knemidocoptes* spp. infection	Right corner of the beak (*angulus oris*)	NA	Excessive bleeding associated with mass regrowth	(14)
*Melopsittacus undulatus*	1 y	m	Kerathoacanthoma and *Knemidocoptes* spp. infection	Left corner of the lower beak (*angulus oris*)	NA	Excessive bleeding associated with mass regrowth	(14)

*NS, not specified.

**y, years; mo, months.

## 2 Case description

**Figure 1:**
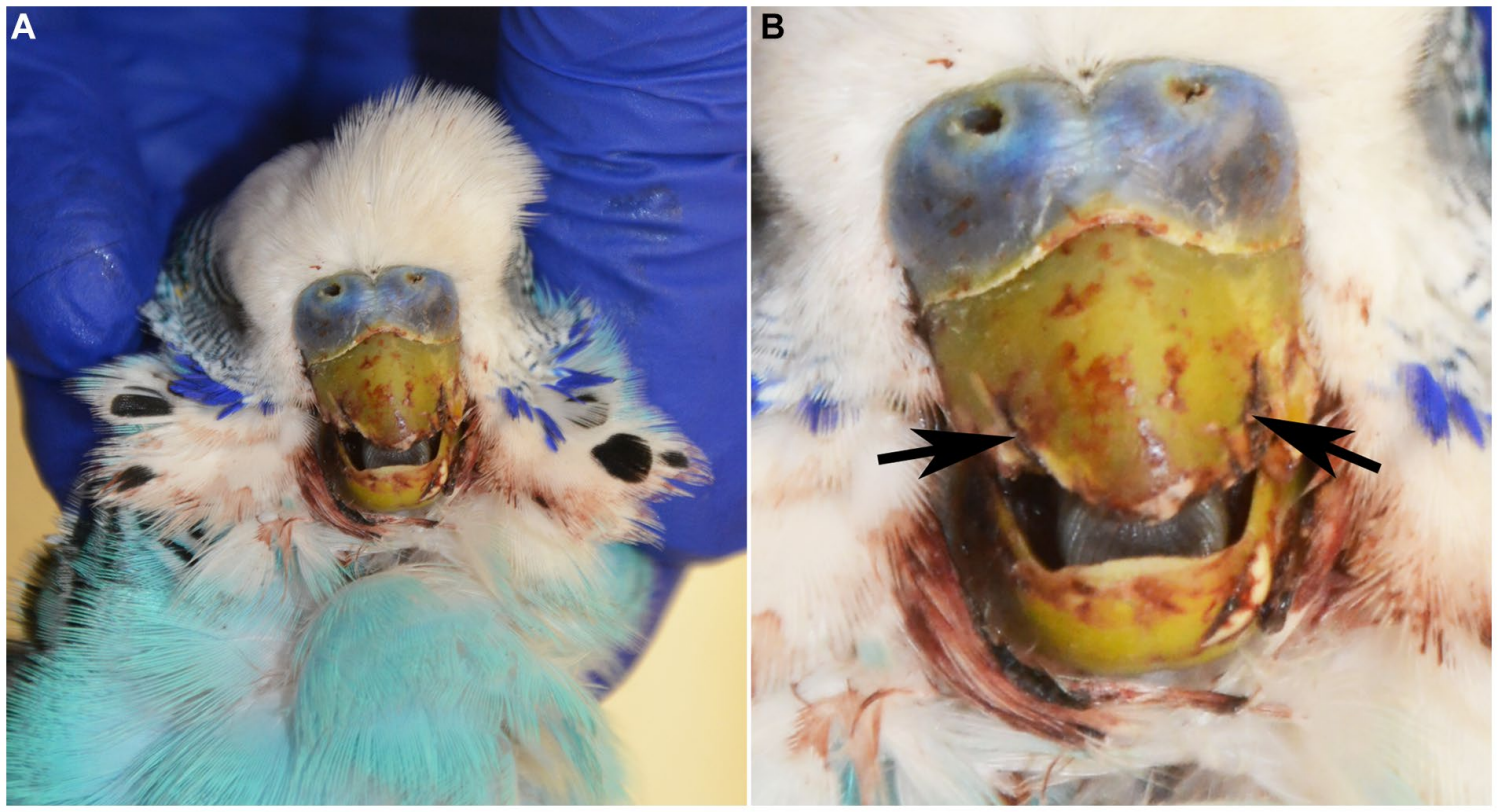
**(A)** 2-year-old male blue budgerigar (*Melopsittacus undulatus*) submitted for post-mortem examination. **(B)** The rostral portion of the rhinotheca was absent with jagged lateral edges (black arrows), incomplete occlusion, and mild hemorrhagic staining of the surrounding feathers.

A 2.5-year-old intact male budgerigar (*Melopsittacus undulatus*) presented to the North Carolina State University Veterinary Teaching Hospital Exotic Animal Services for fracture of the rostral rhinotheca (cornified portion of the upper beak) within the previous 24 h. On physical examination, the bird had pale mucous membranes, mildly increased respiratory effort, dull mentation, ataxia, and suspected seizures involving wing flapping and opisthotonos. The bird died in the hospital within a couple of hours after a physical examination and was submitted for autopsy. On postmortem examination, the rostral portion of the rhinotheca was absent with jagged lateral edges and incomplete occlusion (Figure 1). The feathers surrounding the beak and along the craniodorsal wings were crusted with dried blood. Examination of internal organs was unremarkable.

The head and routine samples of the spleen, heart, liver, lung, trachea, kidney, gastrointestinal tract, testes, brain, spinal cord, and eyes were collected, fixed in 10% neutral buffered formalin, processed routinely, and stained with hematoxylin and eosin. Immunohistochemical staining was also performed on sections of the beak using antibodies specified in Table 2. Negative controls (without the addition of primary antibody) were adequate. Internal positive controls for alpha-smooth muscle actin (α-SMA) (tunica media of arteries), S100 (nerve), and cytokeratin AE1/AE3 (CK AE1/AE3) (nasal epithelium, epidermis) were adequate. Sections of canine cutaneous melanomas were used as positive controls for Melan-A and PNL2. Internal positive controls for vimentin (skeletal muscle, fibrocytes in dermis, and nasal submucosa) were inadequate for three different antibodies, so this immunohistochemical marker was unable to be interpreted in this case. A choanal swab was collected postmortem and submitted for *Chlamydia* spp. PCR at the University of Georgia College of Veterinary Medicine Infectious Diseases Laboratory. *Chlamydia* spp. was not detected in this case.

**Table 2:** Antibodies with source, clone, manufacturer, concentration used, retrieval information, detection method, and positive control used.

Antibody	Host	Source	Clone	Antigen retrieval	Dilution	Chromogen	Autostainer	Internal positive control
α-SMA	Mouse	Biogenex MU128-UC	[1A4]	HIER*	1:100	DAB	Biocare Intellipath, Pacheco, CA	Tunica media of arteries
S-100	Rabbit	Dako Omnis z0311	Polyclonal	HIER*	1:2000	DAB	Biocare Intellipath, Pacheco, CA	Nerve
Cytokeratin AE1/AE3	Mouse	Dako M3515	AE1/AE3	HIER*	1:500	DAB	Biocare Intellipath, Pacheco, CA	Nasal epithelium and epidermis
Melan-A	Mouse	Dako Agilent M7196	A103	HIER*	1:200	DAB	Biocare Intellipath, Pacheco, CA	Canine cutaneous melanoma
PNL2	Mouse	Santa Cruz sc-59306	PNL2	HIER*	1:100	DAB	Biocare Intellipath, Pacheco, CA	
Vimentin	Mouse	Abcam ab80667	Clone V9	HIER*	1:200	DAB	Biocare Intellipath, Pacheco, CA	Skeletal muscle, fibrocytes in the dermis, and nasal submucosa**
	Rabbit	Abcam ab92547	EPR3776	HIER*	1:1000	DAB	Biocare Intellipath, Pacheco, CA	
	Mouse	Dako Agilent M0725	Clone V9	HIER*	1:1000	DAB	Biocare Intellipath, Pacheco, CA	

*Heat-based antigen retrieval was performed using a pressure cooker on a steam setting for 20 min in citrate buffer pH 6 or EDTA/tris buffer pH 9 followed by treatment with 3% hydrogen peroxide.

**Internal positive controls for vimentin (skeletal muscle, fibrocytes in dermis, and nasal submucosa) were inadequate for three different antibodies, so this immunohistochemical marker was unable to be interpreted in this case.

**Figure 2:**
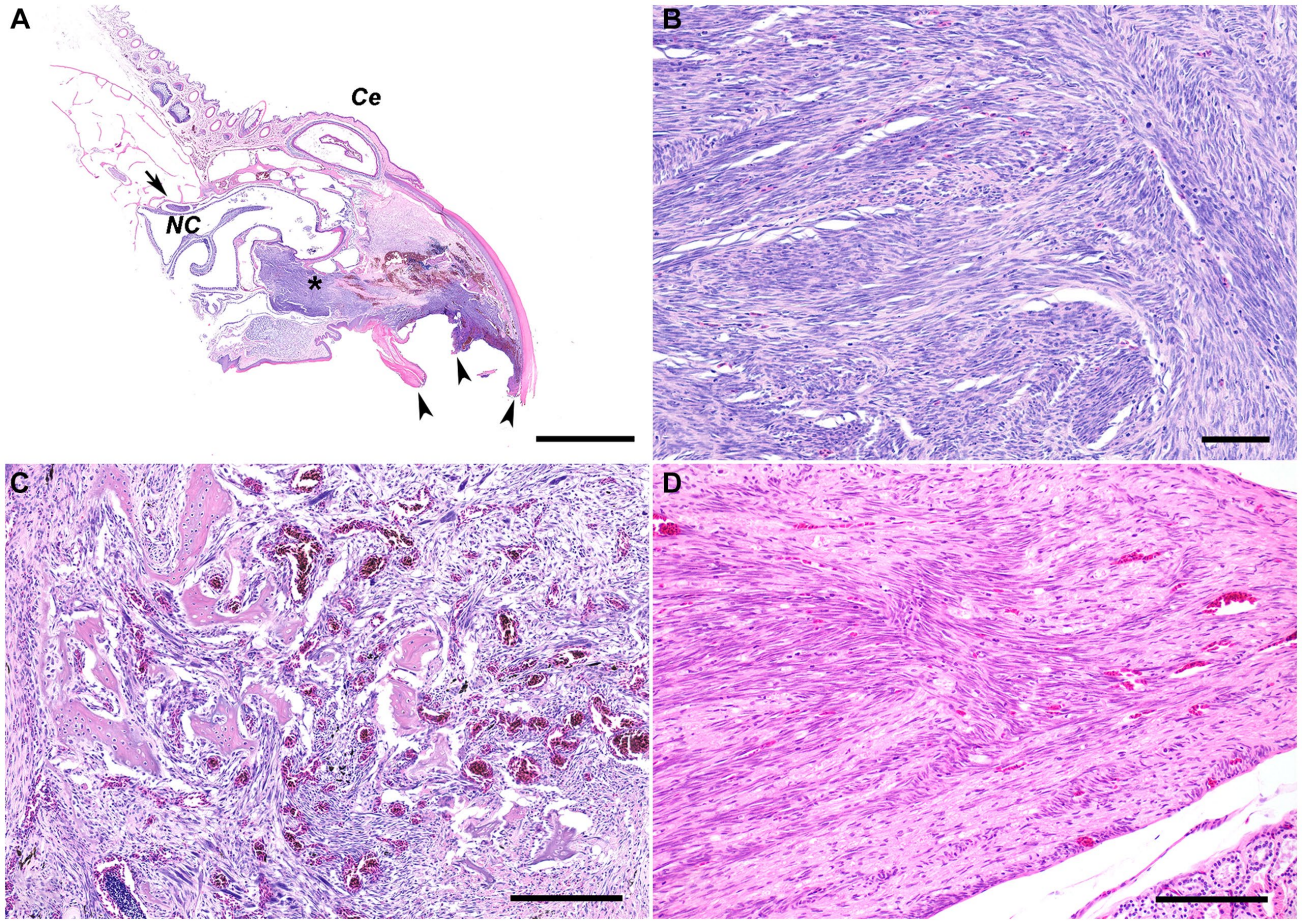
**(A)** Subgross photomicrograph of the rostral rhinotheca. The fractured rostral portion of the beak was observed grossly (arrowheads). Extending from the rostral rhinotheca and effacing the underlying dermis and bone of the premaxilla and maxilla is a non-encapsulated, poorly demarcated, infiltrative neoplastic population with intermixed hemorrhage and areas of necrosis (asterisk). The neoplasm extends caudally with slight ventral compression of the rostral nasal cavity. Caudal to the nasal cavity, a nerve and ganglion are infiltrated by the neoplasm (arrow). *NC*, nasal cavity; *Ce*, cere. H&E stain. Bar = 2 mm. **(B)** The poorly demarcated, non-encapsulated, infiltrative neoplasm consists of densely cellular interweaving streams of well-differentiated spindle cells supported by minimal fibrovascular stroma. H&E stain. Bar = 60 μm. **(C)** The neoplastic spindle cells infiltrate into the medullary cavity of the bone with thinning and loss of the bony trabeculae. Occasional small fragments of necrotic bone are embedded within the neoplastic population and surrounded by macrophages and several multinucleated cells. The remaining trabeculae adjacent to the neoplasm often have scalloped edges with increased numbers of osteoclasts in Howship’s lacunae, consistent with bone resorption/osteolysis. H&E stain. Bar size = 200 μm. **(D)** The nerve and ganglion just caudal to the nasal turbinates are infiltrated by a similar population of neoplastic spindle cells. H&E stain. Bar size = 200 μm.

**Figure 3:**
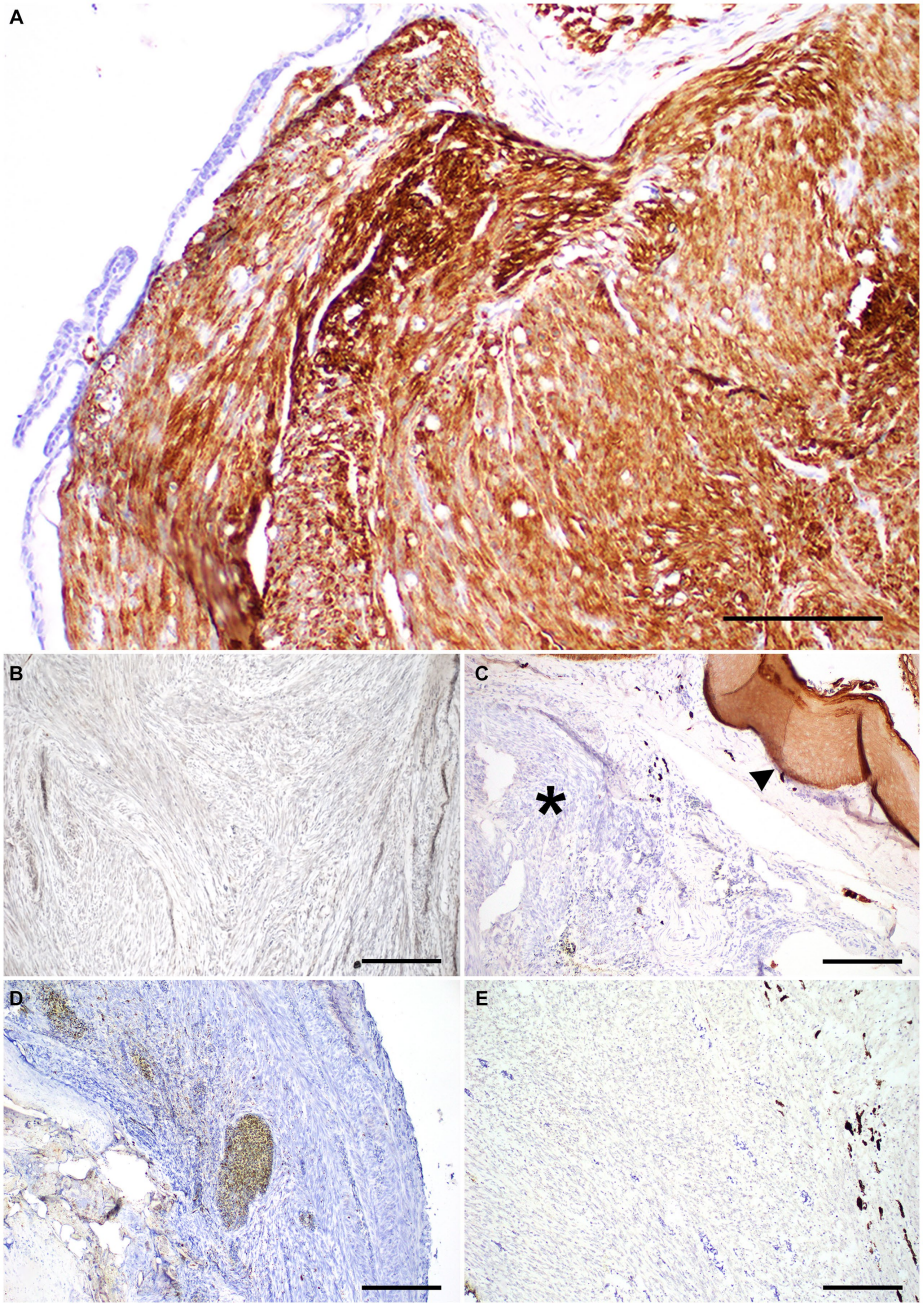
Immunohistochemistry panel on a beak leiomyosarcoma in the rostral rhinotheca of a 2-year-old male budgerigar. **(A)** Neoplastic cells had strong cytoplasmic immunolabeling for α-smooth muscle actin IHC. Bar size = 200 μm. **(B)** Neoplastic cells lacked immunolabeling for S-100 IHC. Bar size = 200 μm. **(C)** Neoplastic cells lack CK AE1/AE3 cytoplasmic immunolabeling (asterisk), normal epithelium is present as internal positive control (arrowhead). Bar size = 200 μm. **(D,E)** PNL-2 and Melan-A immunolabeling was absent in neoplastic cells. Bar size = 200 μm.

Histologic examination of the rostral portion of the upper beak revealed a poorly demarcated, non-encapsulated neoplasm infiltrating the dermis and bones of the premaxilla and maxilla while slightly compressing the rostral nasal cavity (Figure 2A). This neoplasm was composed of densely cellular interweaving streams of spindle cells supported by minimal fibrovascular stroma (Figure 2B). Neoplastic cells had a small amount of eosinophilic cytoplasm with indistinct cell borders and elongated nuclei with densely stippled chromatin. Anisocytosis and anisokaryosis were mild to moderate with 233 mitotic figures in 10400X fields (2.37 mm^2^). Occasional large multifocal areas of coagulative to lytic necrosis were present throughout the neoplasm. Neoplastic infiltration of the medullary cavity of the premaxilla and maxilla was associated with thinning and loss of the bony trabeculae. Occasional small fragments of necrotic bone were embedded within the neoplastic population and surrounded by macrophages and several multinucleated cells (osteoclasts and multinucleated giant cells) (Figure 2C). The remaining trabeculae adjacent to the neoplasm often had scalloped edges with increased numbers of osteoclasts in Howship’s lacunae (bone resorption and osteolysis). The epidermis and cornified material (rhinotheca) along the ventral aspect of the beak tip were absent with exposure of the underlying neoplasm. Along the exposed ulcerated surface of the neoplasm was an abundant hemorrhage, fibrin, eosinophilic necrotic debris, streaming nuclear debris, and moderate numbers of superficial mixed bacterial colonies. A similar population of neoplastic spindle cells infiltrated the trigeminal nerve and ganglion just caudal to the nasal turbinates (Figure 2D) as well as the optic nerve of one eye. Neoplastic metastasis was not observed in intracoelomic organs. Approximately 80% of the neoplastic cells exhibited strong cytoplasmic immunolabeling for α-SMA (Figure 3A) but lacked immunolabeling for S100, CK AE1/AE3, PNL2 and Melan-A (Figures 3B–E).

## 3 Discussion

To better understand the disease process affecting the beak, a summary of the normal anatomy is provided. The beak or bill in birds is commonly known as the rostrum and is formed by the maxillary bones or maxillary rostrum and the mandibular bones or mandibular rostrum covered by their respective horny sheaths (16–18). The upper and lower beak are covered by a keratin structure derived from the epidermis, forming the horny bill or mandibular rhamphotheca (rhinotheca) and mandibular rhamphotheca (gnathotheca) (17, 18). The primary vascular supply to the beak is the common carotid artery and its branches, while the primary innervation of the beak is the trigeminal nerve (18, 19).

Like other bony structures, fractures of the beak may be classified as traumatic or pathologic. Traumatic damage to the beak is most commonly the result of bite wounds from another bird but can also be caused by impact injuries, such as flying into an object (1, 18). Localized disease of the beak and some systemic diseases can decrease the structural integrity of the beak, increasing the chance of significant injury with even minor trauma. Localized disease to the beak may include bacterial or fungal infection, mite infestation (i.e., *Knemidokoptes* spp.), or neoplasia. Systemic diseases that can weaken the structure of the beak include malnutrition (i.e., vitamin A deficiency, metabolic bone disease) and chronic liver disease (18). In this case, neoplastic infiltration of the upper beak likely contributed to the observed fracture of the rostral rhinotheca with secondary superficial bacterial infection. These findings represent a pathologic fracture of the beak in this budgerigar. Evidence of other contributing factors to decreased beak integrity, such as mite infestation, malnutrition, or chronic liver disease was not appreciated by gross or histologic examination in this case.

Neoplastic diseases commonly affecting the beak of birds include fibrosarcoma, squamous cell carcinoma, and malignant melanoma. Fibrosarcomas are considered the most common primary beak tumor in birds, being most prevalent in budgerigars and cockatiels (1). Fibrosarcomas are typically locally aggressive with infrequent metastasis. Like the pathologic beak fracture in this case, there is a description of a beak fracture associated with a neoplasm described in an African Gray Parrot (2). A disseminated fibrosarcoma caused a pathological fracture on the two-thirds of the upper beak and the proximal left tarsometatarsus just below the epiphysis of the bird (2). The authors could not determine the primary origin of the tumor (2). Osteosarcoma and melanoma were considered differential diagnoses however they were ruled out based on the pathological interpretation of each lesion. The evidence of bone formation within the fracture located at the metatarsus was attributed to the callus formation, and the abundant melanin pigmentation present within the cells at the beak lesion was interpreted as remnants of the local tissue invaded by the tumor (2). Another limitation of this case report was the lack of the use of immunohistochemistry. Some authors consider that fibrosarcomas are overdiagnosed because any tumor lacking specific histogenesis, with spindle cell morphology, atypia, and collagenous stroma may be diagnosed as fibrosarcoma (20).

Squamous cell carcinomas are locally invasive with a low metastatic potential (1). Secondary bacterial and mycotic infections are commonly documented (1). Treatment requires aggressive surgical resection, chemotherapy (cisplatin), radiation therapy (cobalt −60), and topical and oral angiogenic therapy with a variable rate of success (5, 21, 22). In this case, the spindle shape of the neoplastic cells along with the lack of immunolabeling for cytokeratin AE1/AE3 ruled out squamous cell carcinoma. Malignant melanomas are reported in several species of birds, and metastasis to the lungs, kidney, liver, spleen, and bone marrow are commonly reported (1, 8–10). Reported treatments include surgical excision, radiation, and angiogenic therapy (8–10). The lack of immunolabeling for Melan-A and PNL2 in the case reported herein suggested that a diagnosis of malignant melanoma was unlikely.

The use of immunohistochemistry as a routine diagnostic tool allows the differentiation of fibrosarcoma from other spindle cell tumors like peripheral nerve sheath tumors, rhabdomyosarcoma, amelanotic malignant melanoma, spindle cell carcinoma, leiomyosarcoma and others (20). PNL2, Melan-A, and Cytokeratin AE1/AE3 are allowed to rule out melanoma and carcinoma. Based on the histologic appearance of the neoplastic population effacing the dermis and bone of the upper beak, additional differentials in this case included peripheral nerve sheath tumor and leiomyosarcoma. Peripheral nerve sheath tumor was considered given the presence of neoplastic infiltration into the cranial nerves and an associated ganglion. However, peripheral nerve sheath tumors are not well-documented in birds. Given the lack of immunolabeling against S-100 (a marker of tumors of neural origin) in this neoplastic population, peripheral nerve sheath tumor was considered less likely (20).

Leiomyosarcomas in birds are most frequently reported in the spleen, gastrointestinal tract, and reproductive tract with occasional reports of cutaneous and subcutaneous leiomyosarcomas (23, 24). Leiomyosarcomas are typically locally invasive with infrequent metastasis to the liver, spleen, bone marrow and thorax (23–27). In this case, positive immunolabeling of the neoplastic cells for α-SMA (a smooth muscle marker), supported a diagnosis of leiomyosarcoma with a primary localization to the beak.

Immunohistochemistry was essential to further characterize the beak neoplasm in this budgerigar with positive immunoreactivity to α-SMA, which is most consistent with leiomyosarcoma (28). The immunolabeling for α-SMA indicates a smooth muscle origin rather than cardiac or skeletal muscle origin (29) and helps to differentiate smooth muscle tumors from fibrosarcomas (30). Leiomyosarcomas and leiomyomas arise from the smooth muscle and consist of interlacing bundles of spindle cells with vesicular nuclei and fibrillary cytoplasm (1). The differentiation between benign and malignant tumors is based on mitotic activity and cellular atypia (1). While most leiomyosarcomas in birds arise from visceral organs, few case reports describe cutaneous and subcutaneous leiomyosarcomas in birds (23). In contrast to visceral leiomyosarcomas, these reports noted a rapid clinical progression of neoplastic disease with metastasis to visceral organs (23). A cutaneous leiomyosarcoma was reported in a 4-year-old female ornamental pigeon (fantail) with multiple metastases to the liver, pancreas, and lungs (29). The final diagnosis was made based on the histologic findings and strong cytoplasmic immunolabeling for α-SMA and vimentin, with a lack of immunolabeling for MyoD1, cytokeratin AE1/AE3, and desmin (29). A cutaneous leiomyosarcoma was described in the cervical region of a 14-year-old male White Carneau pigeon (*Columba livia*) in which the diagnosis was confirmed by positive immunolabeling for desmin and α-SMA and lack of immunolabeling for cytokeratin AE1/AE3, S100, and von Willebrand factor (23). There are also two reports of cutaneous leiomyosarcomas in budgerigars that include one on the caudal coelom of a 6-year-old female and one in the right wing of a 5-year-old female (31). In both cases, the diagnosis was confirmed through immunolabeling for α-SMA and desmin, and no metastases were identified in either case (31, 32). In the latter, osseous metaplasia was identified within the neoplasm (32). This histologic feature was not observed in the case herein. The remnants of bone trabeculae present in this neoplasm were interpreted as the remnants of the maxillary bone rather than osseous metaplasia. Visceral metastasis was not observed in this budgerigar, but local invasion into one optic nerve and the trigeminal nerve and ganglion was present. To our knowledge, leiomyosarcomas have not been previously reported in the beak of birds.

## 4 Conclusion

The limitations of this case are related to the advanced stage of the neoplastic disease at the time of the diagnosis. A more detailed description of the clinicopathological findings associated with this neoplasm could not be performed since the bird died soon after hospitalization. It will remain unclear if neurological signs like dull mentation, ataxia, and suspected seizures involving wing flapping and opisthotonos were a consequence of hypoglycemia, a paraneoplastic syndrome commonly reported in leiomyosarcoma in dogs (28). Additionally, this case report has several diagnostic challenges. Even if beak fracture is commonly seen in birds, it is rarely reported to be secondary to a neoplasm. Fractures of the beak in birds are most attributed to trauma. However, a variety of underlying disease states can act as predisposing factors. On gross examination, there was no evidence of a mass effect, but histologic examination revealed an infiltrative spindle cell neoplasm effacing the bone and dermis of the upper beak, likely contributing to the clinically observed fracture. Based on immunohistochemical labeling of the neoplastic cells with α-SMA and lack of immunolabeling for cytokeratin AE1/AE3, S-100, Melan-A, and PNL-2, this neoplasm was diagnosed as a primary beak leiomyosarcoma. Overt metastasis of this neoplasm was not appreciated grossly or histologically, but there was histologic invasion of the trigeminal nerve and ganglion and one optic nerve. This case report highlights neoplasia as an important differential for fractures of the beak in birds and serves as the first report of primary beak leiomyosarcoma in a bird.

## Data availability statement

The original contributions presented in the study are included in the article, further inquiries can be directed to the corresponding author.

## Ethics statement

Ethical approval was not required for the studies involving animals in accordance with the local legislation and institutional requirements because the owners permitted the post-mortem evaluation. The budgerigar was submitted for routine diagnostic post-mortem examination to the North Carolina State University College of Veterinary Medicine and as a result, was not subject to animal ethics guidelines. Written informed consent was obtained from the owners for the participation of their animals in this study. Written informed consent was obtained from the participant/patient(s) for the publication of this case report.

## Author contributions

BR: Conceptualization, Data curation, Formal analysis, Investigation, Methodology, Writing – original draft, Writing – review & editing. DB: Data curation, Formal analysis, Investigation, Writing – original draft, Writing – review & editing, Conceptualization, Methodology. TN: Conceptualization, Data curation, Formal analysis, Investigation, Methodology, Supervision, Validation, Writing – original draft, Writing – review & editing.
